# The value of renal biopsy in PLA_2_R- antibody-positive patients with proteinuria: impact of additional pathologies on management and prognosis in a Chinese Cohort

**DOI:** 10.3389/fphys.2026.1756029

**Published:** 2026-04-13

**Authors:** Shan Lu, Xiaoyang Wang, Xiaoxue Wang, Mengjiao Zhao, Hong Li, Zhanzheng Zhao

**Affiliations:** Department of Nephrology, the First Affiliated Hospital of Zhengzhou University, Zhengzhou, Henan, China

**Keywords:** Anti-PLA2R antibody, diagnosis, membranous nephropathy, prognosis, renal biopsy

## Abstract

**Introduction:**

The detection of serum anti-phospholipase A_2_ receptor (PLA_2_R) antibodies (SAbs), a method to diagnose membranous nephropathy (MN) that replaces renal biopsy, is becoming increasingly accepted. However, whether SAb detection provides the same clinical value as renal biopsy is uncertain.

**Methods:**

This study aimed to evaluate the value of renal biopsy in the diagnosis, treatment and prognosis of patients with proteinuria who are seropositive for PLA_2_R antibodies (SAb+).

**Results:**

Renal biopsy was performed in 414 SAb+ patients, 284 patients with primary membranous nephropathy (PMN) alone, 11 patients with atypical MN (AMN), 119 patients with PMN and diabetes mellitus (DM) or other pathologies, such as obesity-related glomerulopathy (ORG), renal tubular/interstitial injury (RTI), and ischemic kidney injury (IRI). There was no significant difference in the treatment or prognoses of MN patients with or without additional pathologies/DM. A high estimated glomerular filtration rate (eGFR) was associated with favorable prognosis (P<0.05), but additional pathologies were not significantly associated with it (P>0.05).

**Discussion:**

In conclusion, SAb+ can strongly predict MN (including in patients with DM) in China, but often coexists with additional pathologies. The presence of additional pathologies did not appear to be a significant determinant of management or prognosis. Renal biopsy is still necessary for accurate diagnosis, especially in patients with renal dysfunction.

## Introduction

With the discovery of target antigens, research on membranous nephropathy (MN) has developed rapidly in recent years. There are more than ten known target antigens, accounting for approximately 80–90% of MN cases, of which the serum anti-phospholipase A_2_ receptor (PLA_2_R) is present in approximately 60% of cases (Sethi et al., 2023; Sethi and Fervenza, 2024). The expression of these antigens often leads to unique clinical and pathological phenotypes of MN. Therefore, some studies^1^ have proposed that MN should be classified according to the present target antigens to facilitate patient management and targeted treatment.

The serum anti-phospholipase A_2_ receptor antibody (SAb) detection method has very high specificity and sensitivity (75% and 100%, respectively) for diagnosing MN (McDonnell et al., 2023), and changes in the antibody titer correspond to changes in disease severity (Dong et al., 2019). Studies (Bobart et al., 2019; Wiech et al., 2019; Bobart et al., 2021) have shown that for patients who are seropositive for PLA_2_R antibodies (SAb+) with estimated glomerular filtration rate (eGFR) ≥ 60 ml/min/1.73 m^2^, there are no additional pathologies or information that can alter treatment selection by renal biopsy. The Kidney Disease: Improving Global Outcomes (KDIGO) guidelines state that renal biopsies are not required in SAb+ patients with nephrotic syndrome who have normal renal function but encourage clinicians to perform biopsies when considering the administration of immunosuppressive drugs (Kidney Disease: Improving Global Outcomes (KDIGO) Glomerular Diseases Work Group, 2021). The number of SAb+ patients who do not undergo renal biopsy has significantly increased; if this biomarker is completely reliable, it should provide the same diagnostic and prognostic value as renal biopsy. However, few studies have investigated whether the omission of pathological information in SAb+ patients affects prognosis and treatment selection.

Our previous study (Lu et al., 2024) revealed that the Chinese MN population had a greater proportion and more types of additional pathologies than did the Western population did, which may affect the treatment and prognosis of MN (Wu et al., 2018; Chen et al., 2022). And these studies (Wu et al., 2018; Chen et al., 2022; Lu et al., 2024) have often excluded patients with diabetes, but the proportion was considerable. Therefore, this study aimed to evaluate the value of renal biopsy in the diagnosis, treatment and prognosis of Chinese patients with seropositive PLA_2_R antibodies, including those with DM.

## Methods

SAb+ patients (≥18 years of age) who underwent renal biopsy and received treatment and follow-up at the Department of Nephrology in the First Affiliated Hospital of Zhengzhou University between June 2021 and March 2022 were enrolled in this study. The inclusion criteria were as follows: patients with a pathological diagnosis of MN, including patients with atypical membranous nephropathy (AMN), and primary membranous nephropathy (PMN) alone or with DM/one kind of additional pathology. Patients with secondary DM (steroid-induced diabetes, etc.) and diseases related to secondary MN, such as cancer, autoimmune diseases, M-proteinemia, and infectious diseases, were excluded. To avoid interference with prognostic outcomes, we excluded MN patients with overlapping disease states, such as concurrent DM+IgAN, IgAN+IRI, etc. The Medical Ethics Committee of the First Affiliated Hospital of Zhengzhou University approved the protocol for this retrospective study and waived the requirement for informed consent (protocol code 2024-KY-0869-001, date of approval: 9 September 2024). All methods in our study were conducted in accordance with the relevant guidelines and regulations.

Patient demographic, clinical and pathological data; data on treatment strategies; and data on prognoses were collected. The clinical data collected included kidney function, proteinuria status, hemoglobin level, serum albumin level, etc. Kidney function was assessed by measuring the serum creatinine (SCr) level and determining the eGFR using the Chronic Kidney Disease Epidemiology Collaboration (CKD-EPI) formula. An eGFR<60 mL/min/1.73 m^2^ was considered to indicate renal dysfunction.

Serum anti-PLA_2_R antibody levels were measured using enzyme-linked immunosorbent assay (ELISA, EUROIMMUN Medizinische Labordiagnostika AG, D-23560 Lubeck, Seekamp 31, Germany), and a titer ≥ 14 RU/mL was considered positive (Wu et al., 2018).

Renal biopsy was performed in patients with proteinuria ≥ 0.5 g/24 hours, and biopsies were processed according to standard techniques for light microscopy, immunofluorescence, and electron microscopy (EM). Immunofluorescence detection included IgG (IgG_1-4_), IgM, IgA, C_3_, C_4_, C_1q_, HBsAg, HBcAg, κ, λ, and PLA_2_R, etc, PLA_2_R staining was conducted for all patients. The diagnosis of PMN and other pathologies was made following the KDIGO guidelines for glomerulonephritis (Kidney Disease: Improving Global Outcomes (KDIGO) Glomerular Diseases Work Group, 2021), and the PMN was classified into stages I to IV by characterizing deposits on EM according to the Ehrenreich-Churg staging system (Taguchi et al., 2011). A diagnosis of AMN was made when MN showed cellular proliferation and electron-dense deposits at multiple sites but without a definite etiology. Diabetic kidney disease (DKD) was classified according to the Mogensen staging system. All enrolled patients had pathologically confirmed diagnoses via renal biopsy, and data on additional pathologies were collected.

Patient medical histories were collected, with endpoints including death, dialysis, or complete remission. For the remaining patients, data from the last follow-up were collected as final outcome. The treatment regimens included conservative treatment (CT) with angiotensin II receptor antagonists (ARBs), prednisone (Pred), immunosuppressants such as calcineurin inhibitor/cyclophosphamide/mycophenolate mofetil (CNI/CTX/MMF), and rituximab/obinutuzumab (LT/AT). The definition of a response to treatment included the following: complete remission (CR), usually defined as proteinuria <0.3 g per 24 hours (PCR <300 mg/g); partial remission (PR), defined as proteinuria >0.3 g but <3.5 g per 24 hours or a decrease in proteinuria by ≥50% from the initial value and <3.5 g per 24 hours, with normal or improved renal function compared with before; end-stage renal disease (E), treatment with hemodialysis or peritoneal dialysis; death (D); and no response (N), with no improvement after treatment but no progression to E/D.

### Statistics

SPSS 23.0 software was used for analysis. Categorical variables are presented as frequencies and percentages (%), with group comparisons analyzed using the chi-square test. Normally distributed continuous variables are expressed as the mean ± standard deviation (SD), and the intergroup comparisons were performed via t tests. For nonnormally distributed continuous variables, data are summarized as medians (interquartile ranges, IQRs), and nonparametric tests were used for comparisons. Univariate and multivariate Cox regression analyses were performed to analyze the correlation between the clinical parameters and outcomes. For all variables, the proportional hazard assumption was tested and found to be fulfilled. P ≤ 0.05 was considered to indicate statistical significance.

## Results

### General information

A total of 425 patients were included. Among these patients, the numbers of patients with PMN and TMA (n=2), endothelial cell injury (n=2), or IgA deposition (n=7) were too small to be included in the statistical analysis. The remaining 414 patients all had MN, including 403 patients with PMN (97.3%) and 11 patients with AMN (2.7%). Among the patients with PMN, 284 patients had PMN alone (PMN group, 68.6%), 19 patients had PMN complicated by RTI (PMN-RTI group, 4.6%), 55 patients had DM (13.3%) [44 patients with DM alone (PMN-DM group, 10.6%) and 11 patients had diabetic kidney disease (PMN-DKD group, 2.7%)], 15 patients had IgAN (PMN-IgAN group, 3.6%), 8 patients had IRI (PMN-IRI group, 1.9%), and 22 patients had ORG (PMN-ORG group, 5.3%). A total of 230 patients had hypertension (55.6%), 29 patients with eGFR<60 mL/min/1.73 m^2^.

### Comparison of clinicopathological characteristics, treatments and prognoses between SAb+ patients with PMN alone and those with AMN or PMN with DM or other additional pathologies

Males predominated in all groups except the PMN+DKD group. Compared with the PMN alone group, the PMN+DM and PMN+DKD groups were older; the PMN+DM and PMN+RTI groups had a greater proportion of patients with hypertension; the PMN+DM group had greater anti-PLA2R antibody titers; the PMN+IRI group had lower hemoglobin levels; the PMN+ORG group had greater hemoglobin levels; the PMN+DKD group had lower serum albumin levels and higher cholesterol levels; the PMN+DM, PMN+DKD, PMN+RTI, and PMN+IRI groups had greater levels of proteinuria; the PMN+RTI group had greater SCr levels; the PMN+DM, PMN+RTI, and PMN+IRI groups had lower eGFRs; the PMN+DM and PMN+ORG groups had greater triglyceride levels; and the AMN group had lower triglyceride levels. All differences were statistically significant (P<0.05) (**Table 1**).

**Table 1:** Comparison of clinicopathological characteristics, treatments and prognoses between SAb+ patients with PMN alone and those with AMN or PMN with DM or other additional pathologies.

Total (n=414)	PMN alone (n=284)	PMN+DM (n=44)	PMN+DKD (n=11)	PMN+IgA (n=15)	PMN+RTI (n=19)	AMN (n=11)	PMN+IRI (n=8)	PMN+ORG (n=22)
Basic characteristics								
Gender (male) (n,%)	191 (67.49)	31 (70.45)	4 (36.36)*	10 (66.67)	18 (94.74)*	9 (81.82)	3 (37.50)	17 (77.27)
Age (years)	47.71 ± 12.71	53.09 ± 8.18*	55.82 ± 6.37*	49.13 ± 9.83	46.74 ± 15.50	41.18 ± 16.55	53.50 ± 11.26	46.18 ± 11.56
Hypertension (n,%)	144.00 (50.70)	34 (77.27)*	7 (63.64)	8 (53.33)	15 (78.95)*	5 (45.45)	6 (75.00)	11 (50.00)
Anti-PLA2R titer (RU/mL)	73.00 (32.25,153.18)	113.70 (41,367.40)*	109.00 (41.90,279.20)	46.10 (29.0,87.90)	117.70 (36.00,185.70)	115.3 (35.70,241.00)	69.40 (50.68,142.20)	93 (62.60,176.10)
Hemoglobin (g/L)	133.00 (122.00,146.00)	131 (114.5,146.0)	116.60 (105.00,146.00)	127 (119.0,146.0)	125.00 (109.00,140.00)	135 (128.00,140.00)	98.75 (88.50,141.50)*	140.50 (133.25,158.00)*
Serum albumin (g/L)	25.05 (21.23,29.28)	25.45 (19.38,28.48)	20.70 (16.70,24.70)*	24.70 (17.80,27.70)	20.20 (17.20,25.30)*	28.60 (20.50,30.50)	24.00 (22.35,27.83)	25.95 (23.35,28.20)
Proteinuria (g/24h)	4.45 (2.80,6.97)	6.31 (3.72,8.71)*	9.49 (5.61,12.10)*	2.96 (1.75,5.88)	8.69 (6.49,10.45)*	4.74 (3.89, 11.70)	8.51 (7.72,12.06)*	4.52 (2.00,8.98)
Serum creatinine (umol/L)	70.00 (60.00.82.00)	71.50 (63.0,85.25)	64.00 (48.00,106.00)	70.00 (55.00,84.00)	118.00 (100,173)*	78.00 (65.00, 99.00)	120.0 (47.50,187.25)	78.50 (69.00,93.50)*
eGFR (mL/min/1.73m2)	100.90 ± 18.98	91.66 ± 24.40*	89.30 ± 28.00	99.41 ± 16.09	61.09 ± 31.55*	102.89 ± 26.02	64.40 ± 41.70*	91.67 ± 24.86
Triglycerid (mmol/L)	1.98 (1.37,2.88)	2.46 (1.80,3.62)*	2.79 (2.00,3.41)	1.59 (1.12,2.06)	2.22 (1.76,3.31)	1.20 (0.95,2.32)*	2.00 (1.38,3.48)	3.01 (2.11,4.07)*
Cholesterol (mmol/L)	6.71 (5.23,8.24)	5.92 (4.79,7.37)	8.44 (7.41,9.57)*	6.09 (5.45,8.45)	7.68 (6.19,9.13)	5.72 (4.58,8.52)	6.40 (4.09,8.12)	5.72 (4.95,6.65)*
Therapy								
CT	23 (8.10)	6 (13.60)	2 (18.20)	2 (13.30)	3 (15.80)	3 (27.30)	0 (0.00)*	0 (0.00)
Pred	7 (2.50)	0 (0.00)	0 (0.00)	1 (6.70)	1 (5.30)	0 (0.00)	2 (25.00)*	0 (0.00)
CNI/CTX/MMF	210 (73.90)	34 (77.30)	9 (81.80)	9 (60.00)	12 (63.20)	6 (54.50)	4 (50.00)*	17 (77.30)
LT/AT	41 (14.40)	4 (9.10)	0 (0.00)	3 (20.00)	3 (15.80)	2 (18.20)	1 (12.50)*	5 (22.70)
Follow-up								
PR/CR	224 (78.87)	33 (75.00)	7 (63.64)	12 (80.00)	14 (73.68)	7 (63.64)	4 (50.00)	15 (68.18)
N	46 (16.20)	7 (15.91)	2 (18.20)	3 (20.00)	2 (10.50)	4 (36.36)	1 (12.50)	7 (31.80)
E	7 (2.50)	4 (9.1)	0 (0.00)	0 (0.00)	2 (10.50)	0 (0.00)	2 (25.00)	0 (0.00)
D	7 (2.50)	0 (0.00)	2 (18.20)	0 (0.00)	1 (5.30)	0 (0.00)	1 (12.50)	0 (0.00)

CT, conservative treatment; Pred, prednisone; CNI/CTX/MMF, calcineurin inhibitor/cyclophosphamide/mycophenolate mofetil; LT/AT, rituximab/obinutuzumab; PR/CR, partial remission/complete remission; N, no response; E, end stage of renal disease; D, death.

*: a statistically significant difference compared to PMN alone patients.

In terms of treatment selection, only the PMN+IRI group differed from the other groups (P <0.05), but there was no difference in prognosis among the groups (P>0.05).

### Comparison of the clinicopathological characteristics, treatments and prognoses among SAb+ patients with PMN alone based on different pathological stages

Among the 284 patients with PMN alone, 49 had stage I disease (17.3%), 219 had stage II disease (77.1%), 15 had stage III disease (5.3%), and 1 had stage IV disease (0.3%). The level of serum albumin in the Stage II group was lower than that in the Stage I and Stage IV groups (P <0.05), the eGFR in the Stage III group was greater than that in the Stage I and Stage II groups (P <0.05), and the triglyceride level in the Stage II group was greater than that in the Stage I group (P <0.05) (**Table 2**).

The selection of treatment for and prognosis of PMN were not significantly different among different pathological stages (P>0.05) (**Table 2**).

**Table 2:** Comparison of the clinicopathological characteristics, treatments and prognoses among SAb+ patients with PMN alone based on different pathological stages.

PMN (n=284)	PMN-stage I (n=49)	PMN-stage II (n=219)	PMN-stage III (n=15)	PMN-stage IV (n=1)	P
Basic characteristics					
Gender (male)	31 (63.27)	149 (68.04)	10 (66.67)	1 (100)	0.811
Age (years)	50 (43.50,61.00)	49.00 (39.00,55.00)	31.00 (26.00,54.00)	50.00 (50.00,50.00)	0.015
Hypertension (n,%)	24 (48.98)	111 (50.68)	8 (53.33)	1 (100)	0.784
Anti-PLA2R (RU/mL)	57.90 (31.50,116.00)	76.00 (33.70,172.30)	40.90 (24.60,152.80)	24.90 (24.90,24.90)	0.312
Hemoglobin (g/L)	134.00 (122.50,144.50)	133.00 (122.00,146.00)	127.00 (117.90,149.00)	111 (111,111)	0.541
Serum albumin (g/L)	27.70 (23.35,32.85)	24.30 (21.00,28.60)a	26.20 (21.30, 38.20)	38.80 (38.80,38.80)b	0.002
Proteinuria (g/day)	4.26 (2.40,6.04)	4.58 (2.92,7.35)	3.67 (1.71, 5.61)	1.40 (1.40,1.40)	0.092
Serum creatinine (umol/L)	68.00 (56.65,80.00)	72.00 (61.00,82.00)	65.00 (56.00, 80.00)	88.00 (88.00,88.00)	0.215
eGFR (mL/min/1.73m2)	101.39 (91.67,110.71)	101.94 (90.68,110.31)	116.84 (104.64,132.62)ab	87.85 (87.85,87.85)	0.020
Triglyceride (mmol/L)	1.62 (1.22,2.00)	2.10 (1.49,2.98)a	1.97 (1.33,2.46)	1.35 (1.35,1.35)	0.025
Cholesterol (mmol/L)	6.29 (5.32,8.33)	6.77 (5.25,8.21)	6.89 (4.70,9.14)	4.28 (4.28,4.28)	0.416
Therapy					
CT	7 (14.60)	15 (6.90)	1 (6.70)	0 (0.00)	0.619
Pred	2 (4.20)	5 (2.30)	0 (0.00)	0 (0.00)	
CNI/CTX/MMF	35 (72.90)	164 (75.60)	10 (66.70)	1 (100.00)	
LT/AT	4 (8.30)	33 (15.20)	4 (26.70)	0 (0.00)	
Follow-up					
PR/CR	25 (51.02)	175 (79.91)	14 (93.33)	0 (0.00)	0.796
N	11 (22.40)	33 (15.07)	1 (6.70)	1 (100.00)	
E	1 (2.00)	6 (2.70)	0 (0.00)	0 (0.00)	
D	2 (4.10)	5 (2.30)	0 (0.00)	0 (0.00)	

a, a statistically significant difference compared to PMN-Stage I. b: a statistically significant difference compared to PMN-Stage II.

### Comparison of clinicopathological characteristics, treatments, and prognoses between SAb+ patients with PMN+DM with and without concurrent DKD

In this study, the renal pathology of all SAb+ patients with DM was MN (including the excluded patients). Among the DKD patients, 7 had stage I disease, 1 had stage II disease, and 3 had stage III disease. Compared with the PMN-DM group, there were fewer male patients in the PMN-DKD group (P <0.05) and the cholesterol level was higher (P <0.05). There were no significant differences in treatment selection or prognosis between the DM and DKD groups (P>0.05) (**Table 3**).

**Table 3:** Comparison of clinicopathological characteristics, treatments, and prognoses between SAb+ patients with PMN+DM with and without concurrent DKD.

Characteristics (n=55)	PMN+DM (n=44)	PMN+DKD (n=11)	P
Basic characteristics			
Gender (male)	31 (70.50)	4 (36.40)	0.036
Age (years)	53.09 ± 8.18	55.82 ± 6.37	0.309
Hypertension (n,%)	34 (77.30)	7 (63.60)	0.353
Anti-PLA2R (RU/mL)*	109.00 (41.90, 279.90)	113.70 (41.00, 367.40)	0.923
Hemoglobin (g/L)	128.24 ± 20.88	123.15 ± 24.10	0.486
Serum albumin (g/L)	25.11 ± 6.70	21.46 ± 5.22	0.099
Proteinuria (g/day)	6.83 ± 4.26	9.22 ± 5.24	0.118
Serum creatinine (umol/L)	71.50 (63.00, 85.25)	64.00 (48.00, 106.00)	0.349
eGFR (mL/min/1.73m2)*	91.66 ± 24.40	89.30 ± 28.00	0.781
Triglyceride (mmol/L)	2.76 ± 1.54	3.02 ± 1.58	0.617
Cholesterol (mmol/L)	6.45 ± 2.33	8.57 ± 1.60	0.006
Therapy			
CT	6 (13.60)	2 (18.20)	0.563
Pred	0 (0.00)	0 (0.00)	
CNI/CTX/MMF	34 (77.30)	9 (81.80)	
LT/AT	4 (9.10)	0 (0.00)	
Follow-up			
PR/CR	33 (75.00)	7 (63.64)	0.081
N	7 (15.91)	2 (18.18)	
E	4 (9.09)	0 (0.00)	
D	0 (0.00)	2 (18.18)	

### Analysis of the factors influencing the renal prognosis of SAb+ patients with MN

**Figure 1:**
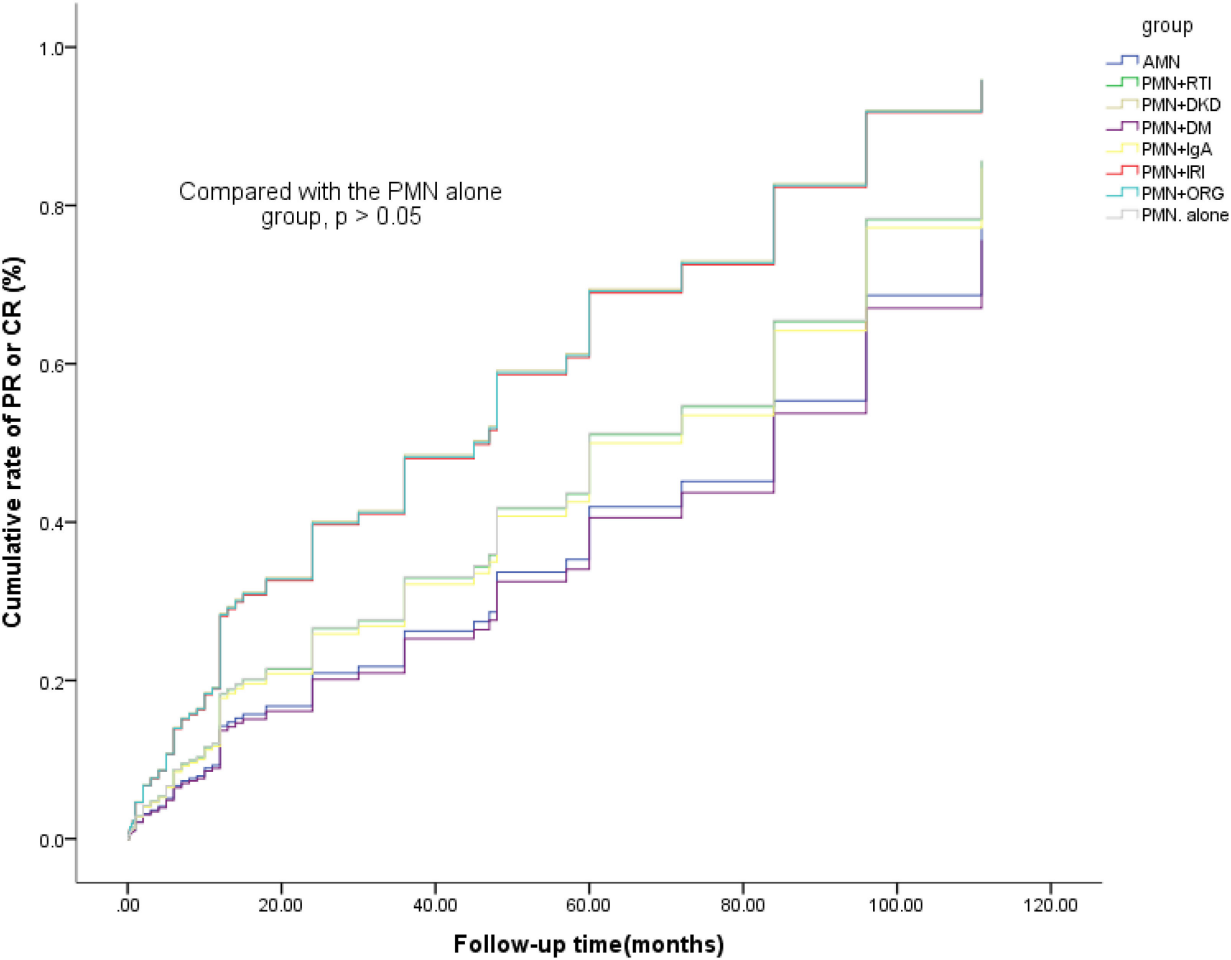
Cumulative incidence rates of PR or CR between SAb+ patients with PMN alone and those with AMN or PMN with DM or other additional pathologies.

Analysis was performed with remission (PR/CR, n=316) and nonremission (N/E, n=87) as the outcome variables, multivariate Cox analysis was performed on non-remission patients to identify factors significantly associated with poor prognosis. The model yielded a likelihood ratio (chi-square) of 831.75, with a p-value of 0.032 (<0.05). And the results revealed that the higher the eGFR was, the lower the noremission rate was. A high eGFR was a protective factor. But the additional pathologies did not affect prognosis (**Table 4**). Furthermore, Kaplan-Meier analysis revealed no significant differences in CR/PR rates between the PMN alone group and the other groups (P > 0.05) (**Figure 1**).

**Table 4:** Analysis of the factors influencing the renal prognosis of SAb+ patients with MN.

Factors	Univariate cox model		Multivariate cox model	
	Hazard ratio (95%CI)	P value	Hazard ratio (95%CI)	P value
Additional Pathologies				
AMN	0.773 (0.274-2.183)	0.627	0.757 (0.263-2.718)	0.606
RTI	1.380 (0.498-3.826)	0.536	0.996 (0.343-2.894)	0.994
DKD	1.646 (0.398-6.802)	0.491	1.652 (0.397-6.864)	0.490
DM	1.018 (0.531-1.952)	0.956	0.725 (0.361-1.456)	0.366
IgAN	0.959 (0.299-3.076)	0.944	0.965 (0.298-3.131)	0.953
IRI	2.444 (0.761-7.844	0.133	1.629 (0.462-5.738)	0.448
ORG	1.695 (0.754-3.812)	0.202	1.639 (0.728-3.690)	0.232
Other Factors				
Anti-PLA_2_R antibody titer	1.001 (1.000-1.002)	0.289	1.000 (0.999-1.001)	0.490
eGFR	0.990 (0.983-0.996)	0.003	0.989 (0.981-0.997)	0.006
Proteinuria/24h	1.017 (0.973-1.063)	0.466	0.987 (0.940-1.035)	0.589
Triglyceride	1.093 (1.000-1.194))	0.050	1.098 (0.988-1.222)	0.084
Cholesterol	1.012 (0.961-1.065)	0.656	0.997 (0.938-1.060)	0.917

## Discussion

With the increasing availability of serum anti-PLA2R antibody tests, more clinicians have accepted that SAb+ patients can be diagnosed as MN without kidney biopsy and that treatment can be formulated according to the antibody titer and clinical manifestations. However, some scholars believe that kidney biopsy is still fundamental for diagnosing MN and characterizing the histological score in terms of the extent of glomerular basement membrane deposits (Murtas et al., 2024), and there is still some uncertainty in the determination of treatment on the basis of the presence and level of antibodies alone (Vink et al., 2023).

In addition to the pathogenicity of the target antigen, activation of the complement system is also involved in the occurrence of MN (Seifert et al., 2023). Murtas, C. et al (Murtas et al., 2024). considered that MN target antigens can be divided into “membrane antigens” and “second-wave” antigens. Second-wave antigens (anti-SOD2, anti-AR, etc.) are involved in the maintenance of the disease and are an independent risk factor for the development of renal failure. These causes cannot all be detected by serology, and renal biopsy can provide more disease-related information; thus, it may be more conducive for treatment selection and prognostic assessment. Our study revealed that Chinese patients with PMN often have additional pathological manifestations, suggesting that the condition is complicated in Chinese patients with MN and that renal biopsy is highly important for an accurate diagnosis.

The avoidance of renal biopsy means that additional pathology cannot be detected. Bobart’s study (Bobart et al., 2019; Bobart et al., 2021) revealed that an additional pathological diagnosis was made in only a small number of patients with MN, mainly those with focal segmental glomerulosclerosis (FSGS); in such patients, the urinary protein level, treatment selection and prognoses were not specific, so it can be disregarded. However, in a Chinese population study (Cheng et al., 2021), patients with PMN and FSGS had more severe clinical and pathological features, which were independent risk factors for poor prognosis. Our study revealed that in Chinese SAb+ patients with PMN, there are many types of additional pathologies, and the proportion of patients with additional pathologies is high; however, unlike in previous studies (Bobart et al., 2019; Bobart et al., 2021), in this study, the number of patients with concomitant FSGS was small, and all were excluded due to loss to follow-up. In our study, the patients with different PMN pathological stages and those with additional pathologies all presented certain differences in clinical manifestations, suggesting that the underlying mechanisms may differ. However, there were no significant differences in treatment selection or prognosis, suggesting that the presence of additional pathologies did not significantly affect treatment or prognosis. Whether this affects long-term prognosis requires more researches.

Among patients with DM and kidney disease, approximately 30–40% have nondiabetic kidney disease (NDKD), with MN being the most common type (Lee et al., 2017; Zhang et al., 2023). A greater proportion of MN patients have DM (Zdravkova et al., 2023). Whether a correlation between DM and MN exists is not clear. Su, Z. et al (Su et al., 2025). conducted a Mendelian randomization (MR) study to show that the genetic predisposition of multiple types of diabetes was correlated with the occurrence of MN and that DM seemed to be a contributing factor for the emergence and development of MN. Zdravkova (Zdravkova et al., 2023) revealed that DM was associated with increased activation of the lectin complement pathway. Therefore, it is speculated that in the context of chronic inflammation, abnormal protein glycosylation and increased activation of the complement pathway in DM can trigger the progression of DN to MN. For such patients, experts (Kidney Disease: Improving Global Outcomes (KDIGO) Glomerular Diseases Work Group, 2021) often recommend renal biopsy to clarify the etiology because comorbid DKD may obscure the origin of proteinuria, and false-positive SAb results have been reported in DKD patients (Dong et al., 2019; Caza and Larsen, 2023; Hoxha et al., 2023). In our study, a high proportion of patients with PMN and DM exist, patients with DM were the oldest and had a higher incidence of hypertension. Elderly patients often have cardiovascular and cerebrovascular diseases and a greater risk of renal biopsy. Our study revealed that all SAb+ patients with DM had PMN, and the SAb titer was greater in these patients, indicating that SAb also has very high specificity in diagnosing PMN in DM patients, and that it is possibly feasible to diagnose PMN by SAb test in patients with DM and proteinuria, that consistent with Zhang H’s study (Zhang et al., 2023). Researchers attributed false-positive SAb results to the use of the ELISA method (Caza and Larsen, 2023; Su et al., 2025), but the results of indirect immunofluorescence assays (IFAs) are usually negative at this time (Bharati et al., 2024). This study used the ELISA method, and no false-positive cases were observed, suggesting that the ELISA method has good reliability, although combining it with IFA would further improve diagnostic accuracy.

One study showed (Xie et al., 2020) that DM is an independent risk factor for the deterioration of renal function in patients with MN, but the presence of DM has no significant effect on the remission rate. Patients with PMN and DM have a poor response to the modified Ponticelli (MP) regimen (Bhadauria et al., 2018). This study revealed that, compared with patients with PMN alone, patients with PMN and DM/DKD presented no significant differences in treatment selection or prognosis, and combined DM/DKD was not a prognostic factor for patients with PMN. Moreover, the current guidelines for DKD do not involve immunosuppressive therapy and thus should not affect the selection of treatment for MN. Therefore, for patients with DM, the SAb test can be considered for diagnosing MN to avoid the risk caused by renal biopsy, and no-steroid therapy should be the first choice. In this study, the pathological grade of DKD was early in most patients, which may be the reason for the lack of effect on prognosis. Consequently, if patients achieve antibody seroconversion but exhibit persistent proteinuria, a renal biopsy should be performed to clarify whether the ongoing proteinuria is caused by DKD to guide subsequent treatments.

The natural progression of PMN is variable, but in the long term, 40–60% of patients with nephrotic syndrome progress to ESRD or die from thrombosis or cardiovascular events (Claudio, 2023). Studies (Yang et al., 2020; Dantas et al., 2023) have reported that the prognostic factors associated with MN include elevated anti-PLA_2_R antibody levels or high baseline levels, an age over 50 years, the level and progression of proteinuria, an elevated Scr level, the presence of glomerulosclerosis and interstitial fibrosis, and the presence of tubular atrophy. Our previous study revealed that thyroid dysfunction was an independent risk factor for poor prognosis in patients with PMN (Wang et al., 2023). A study showed that phenotype-based risk stratification could increase treatment precision and improve outcomes in patients with PLA2R-positive MN (Miao et al., 2025). In this study, the renal prognosis was correlated with eGFR, not with the presence of additional pathological changes, suggesting that renal biopsy is highly important for the precision of diagnosis, but its impact on prognosis may be relatively limited. But in Li Z’s study, the prognostic outcomes of MN in the presence of concurrent IgAN, DKD and FSGS were notably poorer than those of isolated MN (Li et al., 2025). This might be due to the fact that some patients in our study had undergone multiple treatment regimens, which resulted in a better prognosis, and large-sized prospective studies needed to clarify in future. However, Renal biopsy is beneficial for patients with eGFR < 60 mL/min/1.73 m², as it differentiates acute from chronic renal insufficiency, guides treatment, and improves outcomes, and thus should be actively performed.

This study has certain limitations, including its retrospective, single-center design and relatively short follow-up period. As some patients received multiple treatment regimens, we analyzed only the last regimen, meaning initial treatment outcomes are unknown and the impact of previous treatments on the final response cannot be ruled out. Furthermore, some follow-up was conducted by telephone, limiting detailed documentation. Prospective randomized controlled trials are needed to clarify these findings. Second, this study did not include all additional pathologies (e.g., crescents and TMA) or patients with overlapping disease states. According to Pan et al (Pan et al., 2023), crescents are closely associated with MN prognosis and warrant intensive treatment. Glomerulosclerosis and tubulointerstitial fibrosis mostly were mild in our cohort and were not analyzed. Urinary podocytes have been shown to correlate with proteinuria and anti-PLA2R titers (Mella et al., 2020), suggesting that combining these parameters in future studies may enhance the feasibility of non-invasive diagnosis of MN.

In conclusion, this study revealed that anti-PLA_2_R antibody seropositivity is highly predictive of MN in Chinese patients (also suitable for patients with DM), but additional pathologies frequently coexist. There are some differences in the clinical manifestations among PMN patients with different additional pathologies, and renal biopsy plays a critical role in making precise diagnoses and clarifying the underlying etiology. In this study, additional pathologies were not found to significantly influence management or prognosis. This raises the possibility that, for SAb+ patients without evidence of secondary disease, treatment decisions based on clinical parameters (e.g., anti-PLA2R antibody titer, proteinuria, serum creatinine) could be possible. In such cases, renal biopsy might potentially be avoided. Larger prospective studies are required to validate. Nevertheless, as the eGFR influence prognosis, renal biopsy is necessary for patients with renal insufficiency.

## Data Availability

The raw data supporting the conclusions of this article will be made available by the authors, without undue reservation.

## References

[B1] Bhadauria D. Chellappan A. Kaul A. Etta P. Badri V. Kumar Sharma R. . (2018). Idiopathic membranous nephropathy in patients with diabetes mellitus: a diagnostic and therapeutic quandary! Clin. Kidney J. 11, 46–50. doi: 10.1093/ckj/sfx055. PMID: 29423200 PMC5798118

[B2] Bharati J. Waguespack D. R. Beck L. H. (2024). Membranous nephropathy: updates on management. Adv. Kidney Dis. Health 31, 299–308. doi: 10.1053/j.akdh.2024.04.004. PMID: 39084755

[B3] Bobart S. A. De Vriese A. S. Pawar A. S. Zand L. Sethi S. Giesen C. . (2019). Noninvasive diagnosis of primary membranous nephropathy using phospholipase A2 receptor antibodies. Kidney Int. 95, 429–438. doi: 10.1016/j.kint.2018.10.021. PMID: 30665573

[B4] Bobart S. A. Han H. Tehranian S. De Vriese A. S. Roman J. C.L. Sethi S. . (2021). Noninvasive diagnosis of PLA2R-associated membranous nephropathy: a validation study. Clin. J. Am. Soc Nephrol 16, 1833–1839. doi: 10.2215/cjn.05480421. PMID: 34782349 PMC8729491

[B5] Caza T. N. Larsen C. P. (2023). False-positive anti-PLA2R ELISA testing in patients with diabetes mellitus. Kidney Int. 103, 425. doi: 10.1016/j.kint.2022.11.004. PMID: 36681457

[B6] Chen X. Chen S. Li Z. Pan X. Jia Y. Hu Z. . (2022). Correlation of body mass index with clinicopathologic parameters in patients with idiopathic membranous nephropathy. Diabetes Metab. Syndr. Obes. 15, 1897–1909. doi: 10.2147/dmso.s366100. PMID: 35757192 PMC9231685

[B7] Cheng W. Sun L. Dong H. Wang G. Ye N. Wang Y. . (2021). Clinicopathologic characteristic and prognosis in idiopathic membranous nephropathy patients with focal segmental sclerosis lesion: a retrospective observational study. Med. (Baltimore) 100, e23988. doi: 10.1097/md.0000000000023988. PMID: 33545990 PMC7837959

[B8] Claudio P. (2023). Primary membranous nephropathy: an endless story. J. Nephrol 36, 563–574. doi: 10.1007/s40620-022-01461-3. PMID: 36251213

[B9] Dantas M. Silva L. B. B. Pontes B. T. M. Dos Reis M. A. De Lima P. S.N. Moysés Neto M. (2023). Membranous nephropathy. J. Bras. Nefrol 45, 229–243. doi: 10.1590/2175-8239-jbn-2023-0046en. PMID: 37527529 PMC10627124

[B10] Dong D. Fan T. T. Wang Y. Y. Zhang L. Song L. Zhang L. . (2019). Relationship between renal tissues phospholipase A2 receptor and its serum antibody and clinical condition and prognosis of idiopathic membranous nephropathy: a meta-analysis. BMC Nephrol 20, 444. doi: 10.1186/s12882-019-1638-x. PMID: 31791262 PMC6889699

[B11] Hoxha E. Reinhard L. Castedello T. Becker J. U. (2023). False positivity for PLA(2)R1 antibody measured by ELISA in a nephrotic patient with no membranous nephropathy. Kidney Int. 103, 411–415. doi: 10.1016/j.kint.2022.09.011. PMID: 36208829

[B12] Kidney Disease: Improving Global Outcomes (KDIGO) Glomerular Diseases Work Group (2021). KDIGO 2021 clinical practice guideline for the management of glomerular diseases. Kidney Int. 100, S1–s276. doi: 10.1016/j.kint.2021.05.021, PMID: 34556256

[B13] Lee Y. H. Kim K. P. Kim Y. G. Moon J. Y. Jung S. W. Park E. . (2017). Clinicopathological features of diabetic and nondiabetic renal diseases in type 2 diabetic patients with nephrotic-range proteinuria. Med. (Baltimore) 96, e8047. doi: 10.1097/md.0000000000008047. PMID: 28885376 PMC6392986

[B14] Li Z. Zhu S. Zuo K. Le W. Xu F. Wang X. (2025). Clinical manifestations and prognosis of immune-mediated membranous nephropathy concurrent with other glomerulonephritides: a retrospective Chinese cohort analysis. Clin. Nephrol 104, 207–217. doi: 10.5414/cn111627. PMID: 40454526

[B15] Lu S. Xiao J. Liu D. Zhang Y. Dong Y. Zhao Z. (2024). Diagnostic value of renal biopsy in anti-phospholipase A2 receptor antibody-positive patients with proteinuria in China. Sci. Rep. 14, 2907. doi: 10.1038/s41598-024-53445-x. PMID: 38316889 PMC10844597

[B16] McDonnell T. Wu H. H. L. Sinha S. Sinha S. Chinnadurai R. (2023). The role of PLA2R in primary membranous nephropathy: do we still need a kidney biopsy? Genes (Basel) 14, 1343. doi: 10.3390/genes14071343. PMID: 37510247 PMC10380005

[B17] Mella A. Deambrosis I. Mingozzi S. Colla L. Burdese M. Giaretta F. . (2020). Detection of urinary podocytes by flow cytometry in idiopathic membranous nephropathy. Sci. Rep. 10, 16362. doi: 10.1038/s41598-020-73335-2. PMID: 33004982 PMC7530666

[B18] Miao J. Zand J. Vaughan L. Soler M. Encarnación M. M.D. Quintana L. F. . (2025). Clinical phenotypes and renal outcomes in PLA2R-associated membranous nephropathy: a multi-center cohort study with unsupervised cluster analysis. Nephrol Dial Transplant 40, 2309–2318. doi: 10.1093/ndt/gfaf107. PMID: 40577236

[B19] Murtas C. Bruschi M. Spinelli S. Kajana X. Verrina E. E. Angeletti A. . (2024). Novel biomarkers and pathophysiology of membranous nephropathy: PLA2R and beyond. Clin. Kidney J. 17, sfad228. doi: 10.1093/ckj/sfad228. PMID: 38213493 PMC10783244

[B20] Pan Y. Liu L. Chen W. Yang H. Zhang J. Wang Y. (2023). Clinicopathological features and prognosis of primary membranous nephropathy in combination with crescent. Int. Urol Nephrol 55, 1523–1530. doi: 10.1007/s11255-022-03457-1. PMID: 36622536 PMC10185626

[B21] Seifert L. Zahner G. Meyer-Schwesinger C. Hickstein N. Dehde S. Wulf S. . (2023). The classical pathway triggers pathogenic complement activation in membranous nephropathy. Nat. Commun. 14, 473. doi: 10.1681/asn.20213210s1464d. PMID: 36709213 PMC9884226

[B22] Sethi S. Beck L. H. Glassock R. J. Haas M. De Vriese A. S. Caza T. N. . (2023). Mayo Clinic consensus report on membranous nephropathy: proposal for a novel classification. Kidney Int. 104, 1092–1102. doi: 10.1016/j.mayocp.2023.08.006. PMID: 37795587

[B23] Sethi S. Fervenza F. C. (2024). Membranous nephropathy-diagnosis and identification of target antigens. Nephrol Dial Transplant 39, 600–606. doi: 10.1093/ndt/gfad227. PMID: 37863839

[B24] Su Z. Luo Z. Wu D. Liu W. Li W. Yin Z. . (2025). Causality between diabetes and membranous nephropathy: Mendelian randomization. Clin. Exp. Nephrol 29, 227–235. doi: 10.1007/s10157-024-02566-8. PMID: 39375304

[B25] Taguchi T. Oyamada M. Harada T. (2011). Pathology of membranous nephropathy. Nihon Jinzo Gakkai Shi 53, 684–691. 21842601

[B26] Vink C. H. Logt A. V. van der Molen R. G. Hofstra J. M. Wetzels J. F.M. (2023). Antibody-guided therapy in phospholipase A2 receptor-associated membranous nephropathy. Kidney Int. Rep. 8, 432–441. doi: 10.1016/j.ekir.2022.12.003. PMID: 36938074 PMC10014436

[B27] Wang P. Wang S. Huang B. Liu Y. Liu Y. Chen H. . (2023). Clinicopathological features and prognosis of idiopathic membranous nephropathy with thyroid dysfunction. Front. Endocrinol. (Lausanne) 14. doi: 10.3389/fendo.2023.1133521. PMID: 37008916 PMC10060953

[B28] Wiech T. Stahl R. A. K. Hoxha E. (2019). Diagnostic role of renal biopsy in PLA(2)R1-antibody-positive patients with nephrotic syndrome. Mod. Pathol. 32, 1320–1328. doi: 10.1038/s41379-019-0267-z. PMID: 30962506

[B29] Wu X. Liu L. Guo Y. Yang L. (2018). Clinical value of a serum anti-PLA2R antibody in the diagnosis and monitoring of primary membranous nephropathy in adults. Int. J. Nephrol Renovasc Dis. 11, 241–247. doi: 10.2147/ijnrd.s176665. PMID: 30288080 PMC6159797

[B30] Xie Z. Li Z. Dong W. Chen Y. Li R. Wu Y. . (2020). The impact of coexisting diabetes mellitus on clinical outcomes in patients with idiopathic membranous nephropathy: a retrospective observational study. BMC Nephrol 21, 224. doi: 10.1186/s12882-020-01878-7. PMID: 32532223 PMC7291707

[B31] Yang L. Y. Wu Y. S. Dai B. B. Lin S. H. Chen H. Li G. P. . (2020). sPLA2-IB level correlates with hyperlipidemia and the prognosis of idiopathic membranous nephropathy. Curr. Med. Sci. 40, 683–690. doi: 10.1007/s11596-020-2246-5. PMID: 32862379

[B32] Zdravkova I. Y. Tilkiyan E. E. Bozhkova D. M. (2023). Lectin complement pathway and diabetes mellitus in the pathogenesis of membranous nephropathy. Folia Med. (Plovdiv) 65, 597–604. doi: 10.3897/folmed.65.e85472. PMID: 37655378

[B33] Zhang H. Zhu Y. Hu Z. Liu Q. (2023). Serum anti-phospholipase A2 receptor antibody in pathological diagnosis of type 2 diabetes mellitus patients with proteinuria. Sci. Rep. 13, 16608. doi: 10.1038/s41598-023-43766-8. PMID: 37789020 PMC10547755

